# A dual role for miR-210-3p in hypoxia-driven signaling and mitotic regulation in cancer

**DOI:** 10.1186/s43556-026-00530-4

**Published:** 2026-08-10

**Authors:** Jaime San-Juan-Guardado, María Turienzo-Durán, Álvaro Suárez-Priede, Nerea Gómez-Suárez, Candelaria Aguilar-García, Tamara Cubiella, María-Dolores Chiara

**Affiliations:** 1 Health Research Institute of the Principality of Asturias, Av del Hospital Universitario S/N, Oviedo, 33011 Spain; 2 Institute of Oncology of the Principality of Asturias, University of Oviedo, Oviedo, 33006 Spain; 3 Department of Functional Biology, Immunology, University of Oviedo, Oviedo, 33006 Spain

**Keywords:** miR-210-3p, Hypoxia-inducible factor (HIF), Mitotic cell cycle dysregulation, FOXM1, Pseudohypoxia

## Abstract

miR-210-3p is a well-established hypoxia-induced microRNA that is commonly upregulated in a wide range of solid tumors, traditionally linked to mitochondrial repression and hypoxia-inducible factor (HIF) signaling. However, its functional role in cancer remains complex and highly context dependent. Here we perform a comprehensive pan-cancer transcriptomic analysis together with functional assays, revealing that miR-210-3p not only mediates classical hypoxic responses but also amplifies mitotic gene expression through activation of FOXM1. Mechanistically, this effect is shown to be dependent on HIF1α but not on HIF2α and, importantly, it has not been recapitulated by hypoxia alone. Notably, activation of the mitotic program is observed in breast cancer cells but not in head and neck squamous carcinoma models, highlighting a strong degree of context dependency across tumor types. In breast cancer cells, miR-210-3p overexpression enhances FOXM1 phosphorylation, upregulates kinetochore regulators, and induces mitotic defects, correlating with poor prognosis in aggressive tumors. Together, these findings position miR-210-3p as a molecular integrator linking pseudohypoxia to mitotic dysregulation, contributing to tumor aggressiveness by sustaining HIF activity and promoting mitotic stress. This dual functionality reconciles its paradoxical effects on proliferation and highlights its potential as a therapeutic target in cancers characterized by pseudohypoxia and mitotic abnormalities.

**Supplementary Information:**

The online version contains supplementary material available at 10.1186/s43556-026-00530-4.

## Introduction

Hypoxia is a hallmark of solid tumors and a major driver of cancer progression, therapy resistance, and metastasis. Under low oxygen tension, stabilization of hypoxia-inducible factors (HIFs) triggers widespread transcriptional reprogramming that supports metabolic adaptation, angiogenesis, and cell survival under stress. The HIF complex, consisting of oxygen-sensitive HIFα and constitutively expressed HIFβ subunits, orchestrates cellular responses to hypoxia and contributes to abnormal tumor growth [1, 2]. Of the three HIFα isoforms, HIF1α and HIF2α (encoded by *HIF1A* and *EPAS1*, respectively) are the most extensively characterized. HIF1α is broadly expressed in most tumor types, whereas HIF2α is more specifically associated with clear cell renal cell carcinoma, pheochromocytoma, and paraganglioma [3–7].

Among the many HIF-regulated effectors, the microRNA miR-210 has emerged as one of the most consistently induced molecules under hypoxic conditions, earning it the designation of the *“*master hypoxamiR*”* [8]. However, despite extensive evidence supporting its responsiveness to hypoxia, it remains unclear whether miR-210-3p consistently reflects genuine hypoxic signaling across all tumor types, or whether its expression and functions are influenced by tissue-specific and metabolic contexts [9].

Functionally, miR-210 has been implicated in diverse processes, including mitochondrial metabolism, iron-sulfur cluster biogenesis, DNA repair, and angiogenesis [10–15]. Yet, its role in tumor progression remains ambiguous and context dependent. While some studies associate miR-210 upregulation with enhanced proliferation, invasion, and therapy resistance [16–20], others report antiproliferative or pro-apoptotic effects [21–24]. This apparent contradiction suggests that miR-210 may act through distinct, context-specific regulatory networks, possibly influenced by tumor type, stage, or the tumor microenvironment [25, 26]. Similar context-dependent regulatory effects have been described for other non-coding RNAs involved in tumor progression and metabolic control [27, 28].

To date, most mechanistic insights into miR-210 function derive from individual tumor types, limiting our understanding of its broader relevance. For example, in breast cancer, elevated miR-210 levels have been associated with worse clinical outcomes, yet the underlying pathways differ across studies and have not been systematically explored in other tumor types [29–31]. In addition, emerging evidence linked miR-210 to alterations in cell cycle regulation, centrosome amplification, and dysregulation of mitotic gene expression, although these observations have not been integrated into a unified mechanistic framework [32–34]. This complexity underscores the need for a comprehensive pan-cancer investigation of miR-210 expression and its transcriptomic program.

In this study, we present a comprehensive pan-cancer analysis of the mature miR-210-3p expression and its associated gene expression programs, integrating large-scale datasets from The Cancer Genome Atlas (TCGA) and Gene Expression Omnibus (GEO). By identifying both conserved and tumor-specific transcriptional networks linked to miR-210-3p, we reveal a dual role for this microRNA: as a canonical hypoxia effector and a promoter of mitotic gene expression in certain contexts. Our findings challenge the view of miR-210-3p as merely a passive hypoxic marker and instead position it as a key regulator of proliferative reprogramming and mitotic stress in specific cancer types.

## Results

### miR-210-3p expression and transcriptomic interactions across solid tumors

**Fig. 1:**
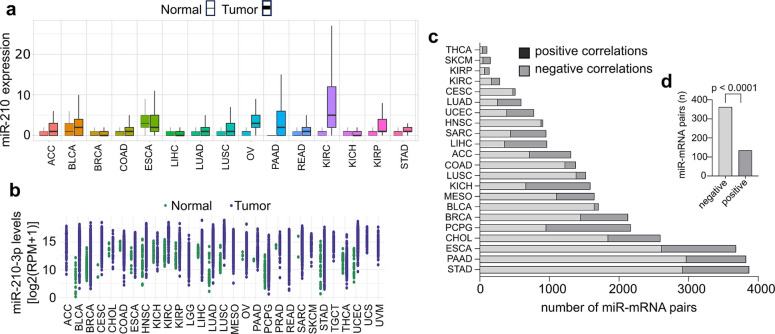
miR-210-3p is recurrently overexpressed in solid tumors and predominantly shows negative correlations with mRNA expression. **a** Boxplots showing miR-210 expression across different cancer types, comparing normal and tumor tissues (p < 0.0001). **b** miR-210-3p expression levels in tumor (purple circle) and normal solid tissues (green circles) from TCGA pan-cancer datasets (log_2_(RPM + 1). **c** Total number of significant positive and negative miR-210-3p mRNA expression correlations across tumor types in TCGA. **d** Bar plot summarizing the total number of miR-210-3p mRNA correlation pairs across all tumor types. ACC: adrenocortical carcinoma, BLCA: bladder urothelial carcinoma, BRCA: breast invasive carcinoma, CESC: cervical squamous cell carcinoma and endocervical adenocarcinoma, CHOL: cholangiocarcinoma, COAD: colon adenocarcinoma, ESCA: esophageal carcinoma, HNSC: head and neck squamous cell carcinoma, KICH: kidney chromophobe, KIRC: kidney renal clear cell carcinoma, KIRP: kidney renal papillary cell carcinoma, LIHC: liver hepatocellular carcinoma, LUAD: lung adenocarcinoma, LUSC: lung squamous cell carcinoma, MESO: mesothelioma, OV: ovarian serous cystadenocarcinoma, PAAD: pancreatic adenocarcinoma, PCPG: pheochromocytoma and paraganglioma, PRAD: prostate adenocarcinoma, READ: rectum adenocarcinoma, SARC: sarcoma, SKCM: skin cutaneous melanoma, STAD stomach adenocarcinoma, TGCT: testicular germ cell tumors, THCA: thyroid carcinoma, UCEC uterine corpus endometrial carcinoma, UCS: uterine carcinosarcoma UVM: uveal melanoma

Comprehensive analysis of 15,648 normal tissues and 40,442 tumor samples from the GEO, GTEx, and TARGET databases revealed a consistent and widespread upregulation of miR-210-3p in the majority of tumor types relative to their normal counterparts (Fig. 1a), underscoring its widespread involvement in cancer biology [35]. Independent validation using TCGA cohort, comprising 8,566 tumor samples from 29 distinct solid tumor types, confirmed that miR-210-3p is recurrently overexpressed in cancer (Fig. 1b).

To delineate the transcriptional programs associated with miR-210-3p activation, we performed a pan-cancer correlation analysis between miRNA and mRNA expression profiles within TCGA. Across tumor types, miR-210-3p expression was significantly more likely to be negatively correlated with mRNA levels (p < 0.0001; Fig. 1c), consistent with its canonical role as post-transcriptional repressor. Integrative cross-tumor analysis identified 362 genes that were inversely correlated with miR-210-3p expression (R < −0.40, p < 0.001) and 134 genes that were positively correlated (R > 0.40, p < 0.001) in at least five tumor types (Fig. 1d; Table S1).

### *ISCU* downregulation supports the identification of conserved miR-210-3p associated transcriptional programs

**Fig. 2:**
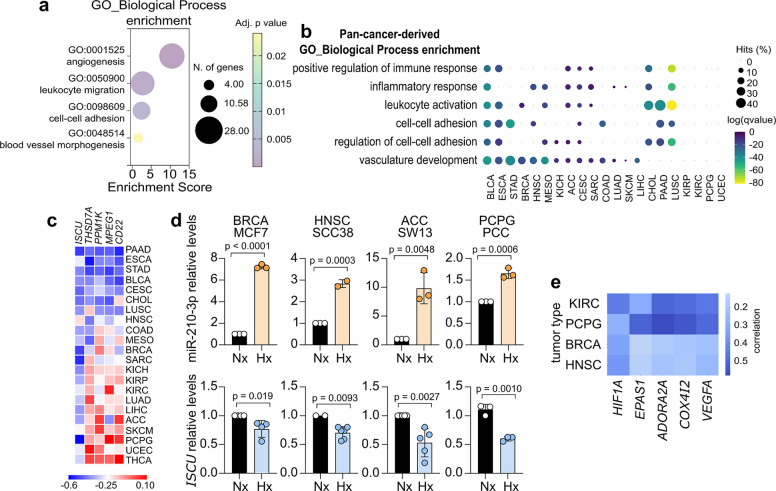
Functional enrichment and validation of genes negatively associated with miR-210-3p across solid tumors. **a** Bubble plot showing Gene Ontology (GO_Biological Process) enrichment for genes negatively correlated with miR-210-3p expression in TCGA pan-cancer analysis. Bubble size indicates the number of genes per category, and color represents adjusted p-value. **b** Dot plot showing enrichment of representative, non-redundant functional GO_Biological Process across individual tumor types. Terms were selected from statistically significant results based on their overlap with predefined functional programs identified in the pan-cancer analysis. Dot size reflects the percentage of negatively correlated genes in each category, and color indicates –log_10_(q-value). **c** Heatmap of Spearman correlation coefficients between miR-210-3p and five conserved predicted targets (*ISCU, THSD7A, PPM1K, MPEG1, CD22*) across cancer types in TCGA. **d** Bar plots showing expression levels of miR-210-3p (top) and *ISCU* (bottom) under normoxic (Nx, 21% O_2_) and hypoxic (Hx, 1% O_2_) conditions in four cancer cell lines: MCF7 (BRCA: breast invasive carcinoma), SCC38 (HNSC: head and neck squamous cell carcinoma), SW13 (ACC: adrenocortical carcinoma), and PCC (PCPG: pheochromocytoma and paraganglioma). Each point represents a biological replicate. Data are shown as mean ± SD (n ≥ 2 independent experiments). **e** Heatmap showing inverse correlations between *ISCU* and canonical HIF2α targets (*ADORA2A, COX4I2, VEGFA*) or HIF genes (*HIF1A, EPAS1*) across selected tumors, suggesting HIF2α-mediated regulation in pseudohypoxic contexts such as KIRC and PCPG. BLCA (bladder urothelial carcinoma), ESCA (esophageal carcinoma), STAD (stomach adenocarcinoma), MESO (mesothelioma), KICH (kidney chromophobe), CESC (cervical squamous cell carcinoma and endocervical adenocarcinoma), SARC (sarcoma), COAD (colon adenocarcinoma), LUAD (lung adenocarcinoma), SKCM (skin cutaneous melanoma), LIHC (liver hepatocellular carcinoma), CHOL (cholangiocarcinoma), PAAD (pancreatic adenocarcinoma), LUSC (lung squamous cell carcinoma), KIRP (kidney renal papillary cell carcinoma), KIRC (kidney renal clear cell carcinoma), UCEC (uterine corpus endometrial carcinoma). Data derived from TCGA tumor datasets; enrichment analysis performed using Metascape. Error bars show mean ± SD

To assess the biological coherence of these associations, we performed a functional enrichment analysis of the 362 inversely correlated genes. The most significantly enriched pathways included vasculature development, blood vessel maturation, immune response, and cell–cell adhesion (Fig. 2a; Table S2), hallmark features of the hypoxic tumor microenvironment. These enrichments were largely consistent across most tumor types (Fig. 2b; Table S3), thereby strengthening confidence in our analytic pipeline. Notably, some exceptions, such as clear cell renal cell carcinomas (KIRC) and paragangliomas/pheochromocytomas (PCPG), showed weaker enrichment. This pattern is consistent with the pseudohypoxic state of KIRC and PCPG, in which HIF signaling is activated under normoxic conditions due to genetic alterations affecting oxygen-sensing or metabolic pathways rather than actual oxygen deprivation [36, 37]. KIRP and UCEC showed a similar pattern indicating that elevated miR-210-3p expression can occur outside the canonical vascular/hypoxic transcriptional context.

To further validate our approach, we intersected the negatively correlated genes with predicted miR-210-3p targets from TargetScan and miRDB, identifying five conserved candidates: *ISCU*, *CD22*, *MPEG1*, *PPM1K*, and *THSD7A*. Among these, *ISCU* (iron–sulfur cluster assembly enzyme) emerged as the most consistently and strongly anti-correlated transcript (Fig. 2c), highlighting it as a robust and recurrent marker of miR-210-3p activity. Consistent with previous studies implicating *ISCU* as a direct target of miR-210-3p and a key mediator of its metabolic effects [10, 11, 38], its identification among the leading-edge genes validated our analytic framework in capturing conserved transcriptional programs associated with miR-210-3p dysregulation.

We next validated the miR-210-3p-*ISCU* axis in representative tumor models. The selected cell lines were chosen to include both tumor types in which the miR-210-3p-*ISCU* interaction has been previously described and tumor types in which this relationship had not been reported, enabling us to assess the conservation of this regulatory axis across distinct cancer contexts. In line with previous reports [10–12, 38], hypoxia-induced miR-210-3p upregulation was consistently accompanied by reduced *ISCU* expression in SCC38 (head and neck squamous cell carcinoma) and MCF7 (breast carcinoma) cells (Fig. 2d). Importantly, we extend these findings to rare tumors such as PCPG and adrenocortical carcinoma (ACC), confirming the repression of *ISCU* in these models. These results highlight the broad conservation of the miR-210-3p-*ISCU* regulatory relationship and support the use of *ISCU* as a functional readout of miR-210-3p activity in diverse cancer types.

While the role of HIF1α in regulating the miR-210-3p–*ISCU* axis is well established [10–12], the contribution of HIF2α remains less well defined. This distinction is particularly relevant for tumors such as PCPG and KIRC, where HIF2α is the predominant hypoxia-responsive transcription factor. To investigate this, we analyzed transcriptomic correlations between *ISCU* and canonical HIF2α target genes (*ADORA2A*, *COX4I2* and *VEGFA*), as well as *EPAS1* and *HIF1A* expression across TCGA datasets. In both PCPG and KIRC, *ISCU* expression showed consistent inverse correlations with *EPAS1* and its downstream targets (Fig. 2e), supporting a role for HIF2α in mediating *ISCU* repression in pseudohypoxic tumors. By contrast, in breast invasive carcinoma (BRCA) and head and neck squamous cell carcinoma (HNSC), *ISCU* levels were inversely correlated with *HIF1A*, consistent with the dominant role of HIF1α in those cancers. Collectively, these findings refine the model of hypoxia-driven *ISCU* regulation and suggest that both HIF1α and HIF2α function as context-dependent mediators of the miR-210-3p-*ISCU* axis across human cancers.

### A novel link between miR-210-3p and mitotic cell division

Having established the robustness of our approach by consistently identifying and experimentally validating *ISCU* as a conserved miR-210-3p target across diverse tumor types, we next investigated whether genes positively correlated with miR-210-3p could reveal additional, previously unrecognized transcriptional programs. Although miR-210-3p is classically viewed as a repressor of mRNA targets, emerging evidence suggests that its activity may also be associated with broader transcriptional changes, possibly due to indirect regulatory effects or shared upstream drivers such as hypoxia-inducible factors [9]. We therefore analyzed the set of positively correlated genes to identify additional pathways that may be co-activated with miR-210-3p and contribute to tumor progression.

**Fig. 3:**
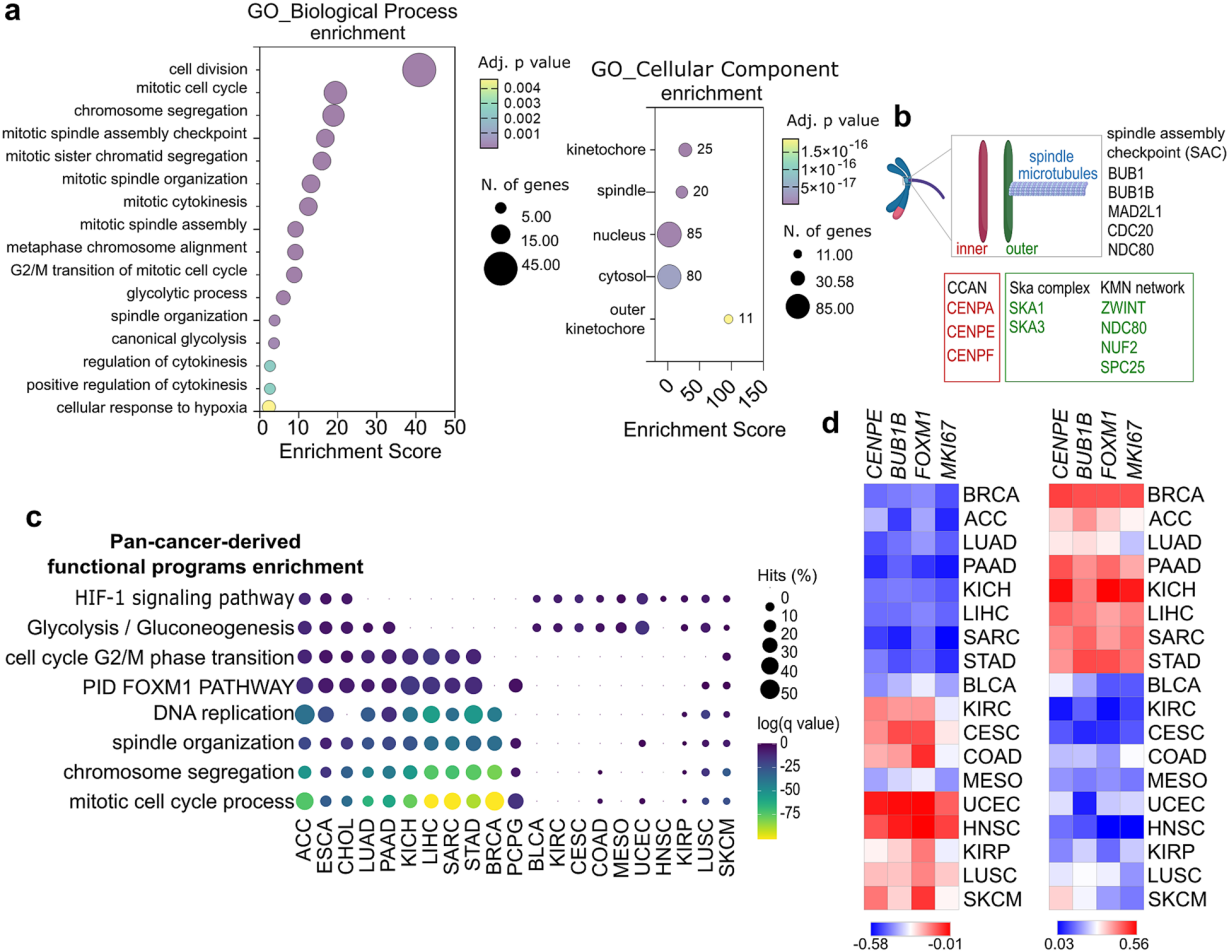
Functional enrichment of genes positively correlated with miR-210-3p expression across solid tumors. **a** Gene Ontology (GO) enrichment analysis of biological processes (left) and cellular components (right). Bubble size indicates the number of genes per category, and color represents adjusted p-value. **b** Schematic overview of kinetochore architecture highlighting genes enriched in the positively correlated gene set, including key components of the spindle assembly checkpoint (SAC) and KMN network. **c** Dot plot showing enrichment of representative, non-redundant functional categories across individual tumor types. Terms were selected from statistically significant results based on their overlap with predefined functional programs identified in the pan-cancer analysis. Functional annotations include GO Biological Process and pathway databases (KEGG, Reactome, and PID). Dot size reflects the percentage of positively correlated genes in each category, and color indicates –log_10_(q-value). **d** Heatmaps showing Pearson correlation coefficients between *ISCU* (left) or miR-210-3p (right) and the indicated mitotic regulators across TCGA tumor types. Data derived from TCGA tumor datasets; enrichment analysis performed using Metascape

Functional enrichment of the 134 genes positively correlated with miR-210-3p expression, identified across TCGA tumor datasets, revealed a significant association with hypoxia-related pathways (Fig. 3a; Table S2: GO:0071456 and related terms) [39], consistent with the widely recognized role of miR-210-3p in the cellular adaptation to low oxygen tension. Strikingly, this analysis also revealed marked enrichment for gene sets involved in cell cycle progression, chromosome segregation, and mitotic division, biological processes not classically linked to miR-210-3p. Complementary cellular component analysis further showed significant overrepresentation of gene products localized to key mitotic structures, including the kinetochore, mitotic spindle, and nucleus (Fig. 3a and b). These included core kinetochore components (*MAD2L1, CDC20, NDC80, CENPE, SKA1, SKA3, KIF2C, ZWINT, BUB1,* and *NUF2*) as well as *FOXM1*, a master transcriptional regulator of mitotic entry and kinetochore gene expression [40] (Table S4).

Remarkably, when tumors were stratified by organ of origin, we observed a distinct pattern of enrichment: some tumors (e.g., kidney chromophobe -KICH-, liver hepatocellular carcinoma -LIHC-, sarcoma -SARC-, stomach adenocarcinoma -STAD-, BRCA, and PCPG) exhibited a predominantly mitotic cell cycle (MCC) transcriptional signature, while others (e.g., bladder urothelial carcinoma -BLCA-, KIRC, cervical squamous cell carcinoma -CESC-, and mesothelioma -MESO-) showed selective enrichment for hypoxic (Hx) programs (Fig. 3c). In tumors with MCC enrichment, *ISCU* expression displayed strong and consistent inverse correlations with mitotic regulators such as *CENPE*, *FOXM1*, and *BUB1B* (Fig. 3d), a pattern not observed in tumors with dominant Hx signatures. *MKI67* correlations further emphasized this divergence. *MKI67* strongly tracks with miR-210-3p (and inversely with *ISCU*) in MCC-enriched tumors, but not in Hx enriched tumors (Fig. 3d). Thus, the miR-210-3p-high/*ISCU-*low axis can associate either with hypoxic remodeling or with mitotic transcriptional programs depending on tumor context.

This observation raises the possibility that, in a subset of cancers, miR-210-3p may function not only as a classical hypoxia effector but also as a contributor to the activation of mitotic gene expression programs. To test this hypothesis, we selected breast cancer, a tumor type with pronounced MCC enrichment, for mechanistic validation.

### Direct role of miR-210-3p in the mitotic program

To determine whether miR-210-3p directly activates the mitotic transcriptional program, we transfected MCF7 breast cancer cells with either miR-210-3p mimics or a non-targeting miRNA control to dissect the specific effects of miR-210-3p from those driven by the broader hypoxic response. *BUB1B* and CENPE were selected as representative readouts of the mitotic program based on their consistent upregulation in tumors exhibiting activation of a FOXM1-driven cell division network, as previously described [40–42], and their established roles in spindle assembly checkpoint control and chromosome alignment, respectively [43, 44].

**Fig. 4:**
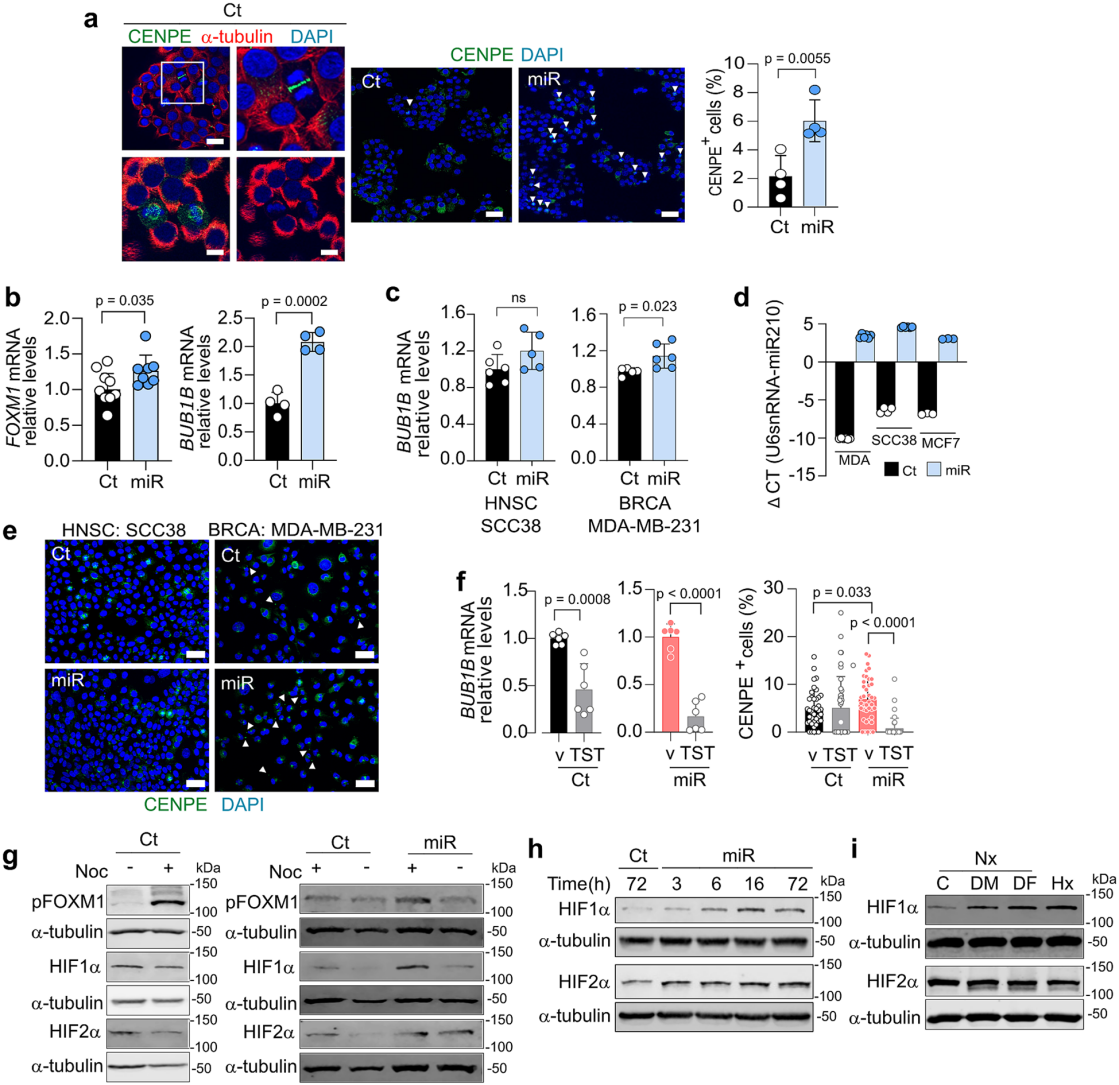
miR-210-3p enhances mitotic gene expression and promotes HIFα accumulation. **a**, **e** Representative immunofluorescence images of CENPE (green) and α-tubulin (red) in MCF7 (**a**) or SCC38 and MDA-MB-231 cells (**e**) transfected with control (Ct) or miR-210-3p mimic (miR). Arrowheads indicate CENPE-positive mitotic cells and CENPE foci in panels a and e, respectively. Nuclei are stained with DAPI (blue). Scale bars: 20 μm (left) and 50 μm (right) in panel a, and 50 μm in panel e. HNSC: head and neck squamous cell carcinoma, BRCA: breast carcinoma. Quantification of CENPE-positive MCF7 cells is shown on the right in panel a. **b** RT-qPCR analysis of *FOXM1* and *BUB1B* mRNA in Ct and miR MCF7 cells. **c** RT-qPCR quantification of *BUB1B* mRNA in the indicated cell lines transfected with control (Ct) or miR-210-3p mimic (miR). **d** RT-qPCR analysis of miR-210-3p expression levels following transfection with control (Ct) or miR-210-3p mimics (miR) in the indicated cell lines, shown as ΔCT values relative to U6 snRNA. Higher ΔCT values indicate higher miR-210-3p expression. **f** RT-qPCR quantification of *BUB1B* mRNA and percentage of CENPE-positive cells in Ct and miR MCF7 cells treated with DMSO vehicle (v) or thiostrepton (TST; 2 μM, 24 h). **g** Left: Western blot analysis of phosphorylated FOXM1 (pFOXM1), HIF1α, and HIF2α in Ct and miR MCF7 cells treated ( +) or not (-) with nocodazole (Noc, 1 μM) for 14 h. Right: Cells were treated with nocodazole for 14 h and either collected immediately ( +) or released into drug-free medium (-) for an additional 2 h prior to lysis. **h** Time-course western blot showing HIF1α and HIF2α accumulation in Ct and miR MCF7 cells from 3 to 72 h post-transfection. **i** Western blot analysis of HIF1α and HIF2α in cells exposed for 24 h to hypoxia (Hx; 1% O₂) or to DMOG (DM; 1 mM), or deferoxamine (DF; 150 μM) under normoxic conditions (Nx, 21% O₂). α-tubulin was used as loading control. Each dot represents an independent biological replicate. Data are shown as mean ± SD (n ≥ 2 independent experiments). Statistical comparisons were made using unpaired two-tailed Student’s t-test. ns, not significant

Overexpression of miR-210-3p significantly upregulated key mitotic regulators: the proportion of CENPE-positive cells increased ~ fourfold (p = 0.005), *BUB1B* mRNA levels doubled (p = 0.0002), and *FOXM1* expression showed a modest but significant increase (1.26-fold, p = 0.035) (Fig. 4a and b). To assess the generalizability of these findings, we extended our analysis to an additional breast cancer cell line (MDA-MB-231) and to a head and neck squamous carcinoma cell line (SCC38). In MDA-MB-231 cells, miR-210-3p overexpression resulted in a significant increase in *BUB1B* mRNA levels (p = 0.023), consistent with the response observed in MCF7 cells. In contrast, no significant induction of *BUB1B* was detected in SCC38 cells under the same conditions (Fig. 4c), despite comparable levels of miR-210-3p expression (Fig. 4d). Consistent with these data, no changes in the percentage of CENPE-positive cells were observed in SCC38 cells (Fig. 4e). In MDA-MB-231 cells, both control and miR-210-3p-overexpressing cells showed widespread perinuclear CENPE accumulation. However, miR-210-3p overexpression increased the fraction of cells displaying an additional intense cytoplasmic CENPE focus (22% vs ~14.5% in controls; > 200 cells quantified) (Fig. 4e).

These observations prompted us to further investigate the molecular mechanism underlying miR-210-3p-mediated mitotic activation in MCF7 cells. In this model, miR-210-3p potentiated mitotic signaling, possibly through FOXM1 activation. To test this hypothesis, we treated cells with the FOXM1 inhibitor thiostrepton (TST). TST suppressed both the percentage of CENPE-positive cells and *BUB1B* mRNA levels, with significantly stronger effects in miR-210-3p-overexpressing cells than in controls (Fig. 4f), supporting a model in which miR-210-3p enhances FOXM1-dependent transcription.

Because FOXM1 is activated via phosphorylation during mitotic entry, we next examined FOXM1 activation by measuring its phosphorylated form (pFOXM1) under conditions of nocodazole-induced mitotic arrest, which enriches the cell population in mitosis. Nocodazole treatment led to pFOXM1 accumulation in control cells as expected; however, pFOXM1 levels were markedly higher in miR-210-3p-overexpressing cells (Fig. 4g; Fig. S1), indicating that miR-210-3p amplifies FOXM1 activation at a critical mitotic checkpoint.

**Fig. 5:**
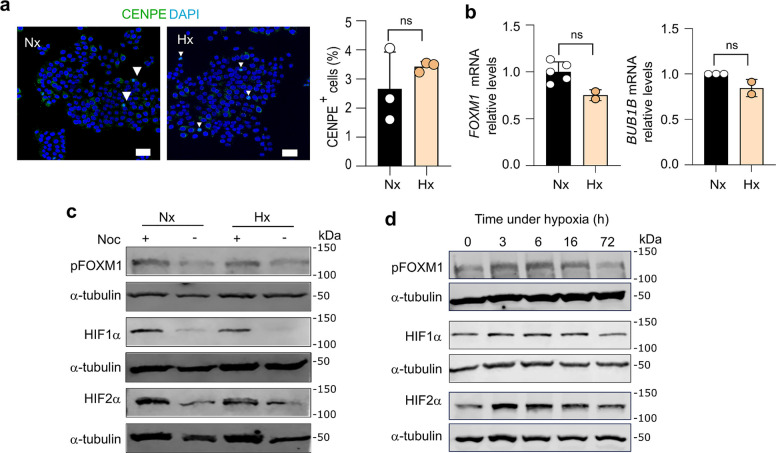
Hypoxia does not activate mitotic gene expression. **a** Representative immunofluorescence images of CENPE (green) in MCF7 cells cultured under normoxia (Nx, 21% O_2_) or hypoxia (Hx, 1% O_2_) for 24 h. Nuclei are stained with DAPI (blue). Arrowheads indicate CENPE-positive mitotic cells. The percentage of CENPE-positive mitotic cells is quantified on the right. Scale bars: 50 μm. **b** RT–qPCR quantification of *FOXM1* and *BUB1B* mRNA levels under Nx or Hx conditions for 24 h. Each dot represents an independent biological replicate. **c** Western blot analysis of phosphorylated FOXM1 (pFOXM1), HIF1α, and HIF2α in Nx or Hx cells treated with nocodazole (Noc, 1 μM) for 14 h and either collected immediately (+) or released into drug-free medium (-) for an additional 2 h prior to lysis. **d** Time-course western blot showing pFOXM1, HIF1α, and HIF2α protein levels in cells exposed to 1% O₂ for the indicated times. α-tubulin was used as loading control. Data are shown as mean ± SD (n ≥ 2 independent experiments). ns, not significant

Given the known interplay between miR-210-3p and hypoxia signaling, we next assessed HIF stabilization following miR-210-3p overexpression. Both HIF1α and HIF2α protein levels increased in a time-dependent manner after transfection and remained elevated up to 72 h, reaching levels comparable to those induced by 24 h hypoxia (1% O₂) or treatment with the HIF stabilizers DMOG and deferoxamine (Fig. 4h and i; Fig. S1). Notably, this sustained HIF1α accumulation contrasts with the transient stabilization observed under hypoxic conditions, where HIF1α levels declined at later time points (Fig. 5d; Fig. S1). However, hypoxia alone failed to replicate the mitotic phenotype observed with miR-210-3p. Exposure of MCF7 cells to 1% O₂ for 24 h did not induce *FOXM1* or *BUB1B* expression, had no significant effect on CENPE levels (Fig. 5a and b), and did not enhance pFOXM1 during mitotic arrest (Fig. 5c; Fig. S1). Although a transient rise in pFOXM1 was detected during early hypoxia (3–16 h), levels returned to baseline by 16 h (Fig. 5d; Fig. S1), indicating a short-lived mitotic response to low oxygen.

**Fig. 6:**
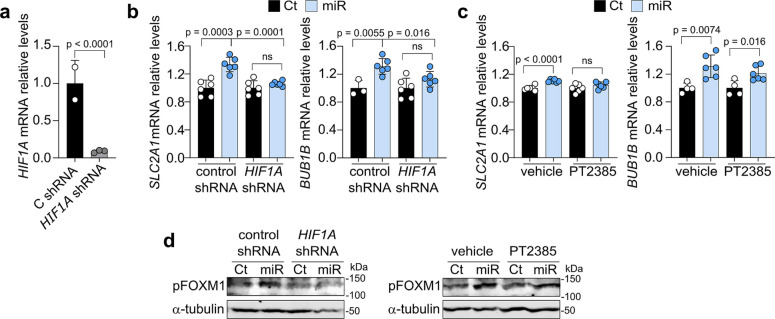
miR-210-3p-induced FOXM1 activation is dependent on HIF1α but not HIF2α. **a** RT–qPCR analysis confirming efficient silencing of *HIF1A* in MCF7 cells transduced with control (C shRNA) or *HIF1A*-targeting shRNA. **b** RT–qPCR analysis of *SLC2A1* (left) and *BUB1B* (right) mRNA levels in control and *HIF1A*-silenced cells following transfection with control (Ct) or miR-210-3p mimics (miR). **c** RT–qPCR analysis of *SLC2A1* (left) and *BUB1B* (right) mRNA levels in cells transfected with Ct or miR-210-3p mimics and treated with vehicle or the HIF2α inhibitor PT2385. **d** Western blot analysis of phosphorylated FOXM1 (pFOXM1) in control and *HIF1A*-silenced cells (left), and in cells treated with vehicle or PT2385 (right), transfected with Ct or miR-210-3p mimics. α-tubulin was used as a loading control. Each dot represents an independent biological replicate. Data are shown as mean ± SD (n ≥ 2 independent experiments). Statistical significance was determined using unpaired two-tailed Student’s t-test. ns, not significant

To further investigate the mechanism underlying FOXM1 activation by miR-210-3p, we assessed the contribution of specific HIF isoforms. Silencing HIF1α in MCF7 cells using shRNA abrogated the induction of the canonical HIF target gene *SLC2A1* upon miR-210-3p overexpression, indicating that miR-210-3p enhances HIF1α transcriptional activity under these conditions (Fig. 6a and b). Strikingly, the miR-210-3p-induced increase in pFOXM1 was abolished in HIF1α-deficient cells (Fig. 6d; Fig. S1). Similarly, upregulation of the FOXM1 target gene *BUB1B* was no longer observed upon miR-210-3p overexpression in the absence of HIF1α (Fig. 6b). In contrast, pharmacological inhibition of HIF2α with PT2385 had no significant effect on pFOXM1 levels or *BUB1B* expression (Fig. 6c and d; Fig. S1), suggesting that miR-210-3p-mediated FOXM1 activation is primarily dependent on HIF1α rather than HIF2α.

Together, these findings support a model in which miR-210-3p promotes FOXM1 activation through HIF1α-dependent signaling, rather than through a direct regulatory interaction or a general hypoxic response.

### miR-210-3p overexpression disrupts mitotic fidelity and impairs cell proliferation

**Fig. 7:**
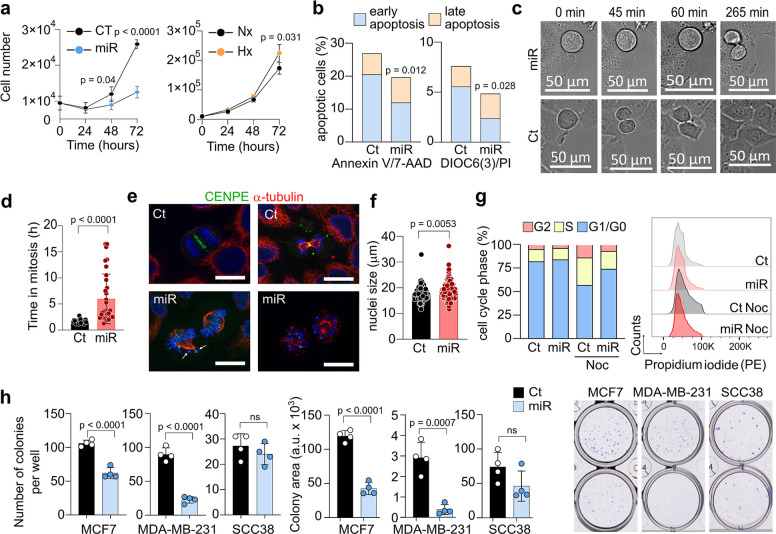
miR-210-3p induces mitotic delay and causes mitotic defects. **a** Growth curves of MCF7 cells transfected with control (Ct) or miR-210-3p mimics (miR) (left), and cells cultured under normoxia (Nx, 21% O₂) or hypoxia (Hx, 1% O₂) (right), monitored over 72 h. **b** Quantification of apoptotic cell populations in Ct and miR cells, assessed by Annexin V/7-AAD (left) and DiOC6(3)/PI staining (right). Bars represent the mean percentage of cells undergoing early apoptosis (blue) or late apoptosis (orange). **c** Representative time-lapse images of dividing Ct and miR-210-3p-overexpressing (miR) cells. Time after mitotic entry is indicated. **d** Quantification of mitotic duration in Ct and miR cells based on live-cell imaging. **e** Immunofluorescence staining of mitotic Ct and miR cells using antibodies against CENPE (green) and α-tubulin (red), with DAPI (blue) to label DNA. White arrows highlight aberrant DNA localization outside the metaphase plate, indicative of mitotic defects. Scale bars: 20 μm. **f** Quantification of nuclear size in Ct and miR cells. **g** Bar plot showing cell cycle distribution of Ct or miR cells treated with or without nocodazole (Noc; 1 μM, 14 h) and released into drug-free medium. Representative DNA content histograms are shown on the right. **h** Colony formation assays in the indicated cell transfected with Ct or miR-210-3p mimics. Left, quantification of the number of colonies and colony area per well; right, representative images of crystal violet–stained colonies. Data are presented as mean ± SD (n ≥ 2 independent experiments). Statistical significance was determined using two-tailed unpaired Student’s t-tests. ns, not significant

To evaluate the functional consequences of miR-210-3p overexpression on cell proliferation and mitotic dynamics, we performed proliferation assays and time-lapse microscopy in MCF7 cells. Despite its ability to activate mitotic gene expression, miR-210-3p overexpression significantly impaired cell growth compared to control cells. In contrast, exposure to hypoxic conditions (1% O₂) enhanced proliferation, underscoring a divergence between the effects of hypoxia and miR-210-3p (Fig. 7a).

To determine whether this reduction in proliferation was attributable to increased cell death, we performed Annexin V/7-AAD and DiOC6(3)/PI analyses. miR-210-3p overexpression did not result in a substantial increase in apoptotic cell death, indicating that the impaired proliferative capacity is not primarily driven by apoptosis (Fig. 7b).

Live-cell imaging revealed a pronounced mitotic delay in miR-210-3p-overexpressing cells, with a substantial fraction failing to complete cytokinesis within the imaging window (Fig. 7c and d; Supplementary video 1 and 2). Immunofluorescence staining of CENPE and α-tubulin further revealed reproducible mitotic abnormalities, including chromosome misalignment and aberrant spindle architecture (Fig. 7e). In addition, miR-210-3p-transfected cells exhibited a significant increase in nuclear size compared to controls, suggesting altered chromatin organization or cell cycle regulation (Fig. 7f). These defects were consistently observed across independent experiments.

To further dissect the basis of the proliferation defect, we performed cell cycle profiling by flow cytometry. miR-210-3p-overexpressing cells showed accumulation in the G1/G0 phase and a concomitant reduction in the S-phase population, indicative of impaired G1–S transition. Following nocodazole treatment, which arrests cells at the G2/M boundary, both control and miR-210-3p-expressing cells accumulated in G2/M; however, the degree of accumulation was lower in miR-210-3p-expressing cells, consistent with reduced cycle progression toward mitosis (Fig. 7g). These data indicate that miR-210-3p imposes a dual block on proliferation by slowing G1-S progression and compromising mitotic fidelity, ultimately impairing cell division and promoting nuclear abnormalities.

To further assess the long-term proliferative potential of miR-210-3p-expressing cells, we performed colony formation assays. miR-210-3p overexpression significantly reduced colony formation in MCF7 and MDA-MB-231 cells, whereas SCC38 cells were largely unaffected (Fig. 7h), consistent with a context-dependent response. These findings indicate that miR-210-3p not only disrupts mitotic fidelity but also limits the long-term proliferative capacity in breast cancer models.

### miR-210 expression is associated with poor clinical outcome in multiple cancer types

**Fig. 8:**
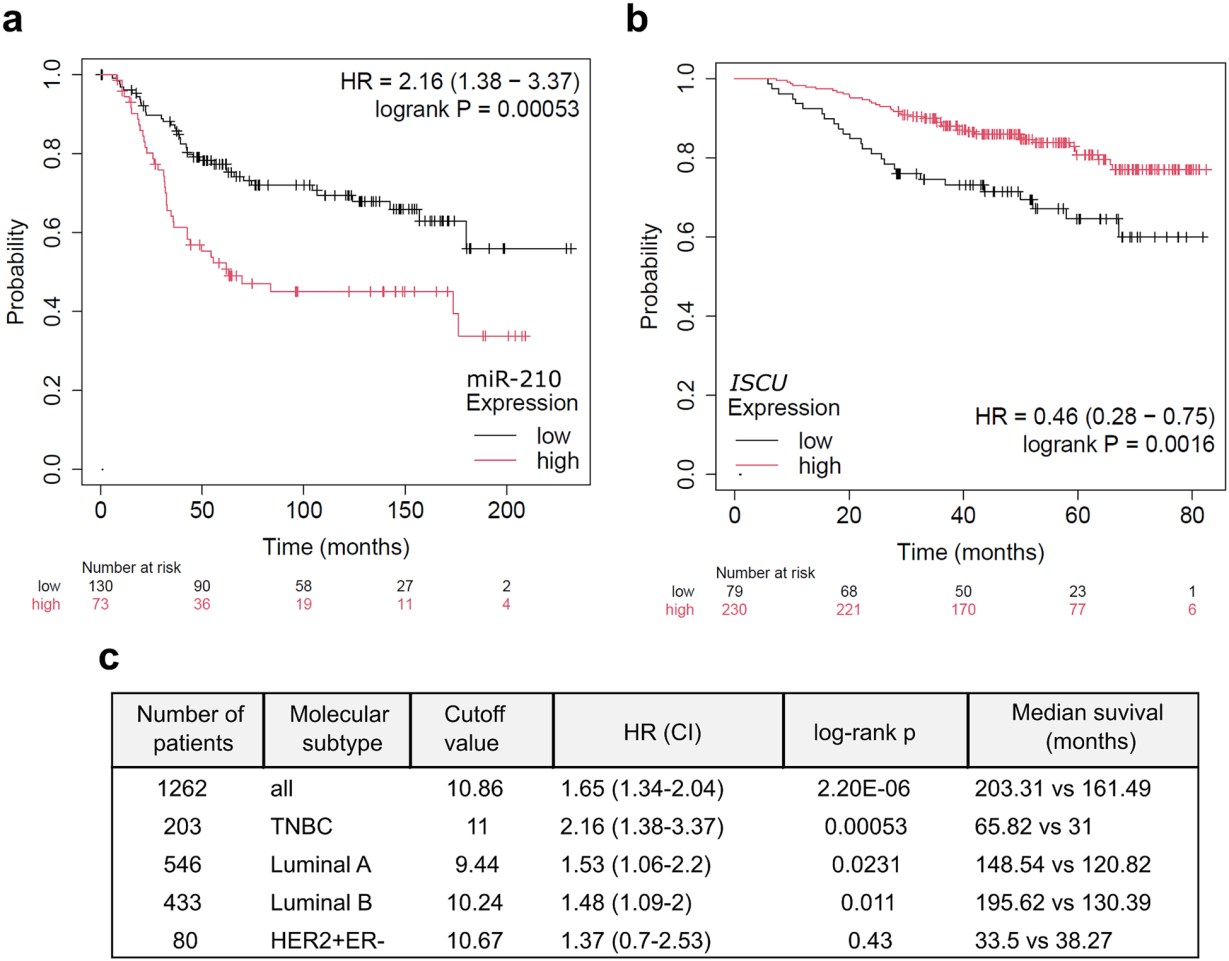
Prognostic value of miR-210 and *ISCU* expression in breast cancer. **a**–**b** Kaplan–Meier survival curves of overall survival stratified by miR-210 (**a**) and *ISCU* (**b**) expression levels. High miR-210 expression is associated with poor prognosis in triple-negative breast cancer (TNBC) patients from the METABRIC cohort. Low *ISCU* expression correlates with poor prognosis in basal-like breast cancer. **c** Summary table showing hazard ratios (HR), log-rank p-values, and median survival times for miR-210 across all breast cancer patients and molecular subtypes in METABRIC, as determined using the KM Plotter tool

To evaluate the prognostic significance of miR-210-3p, we analyzed overall survival data from the publicly available METABRIC cohort using the KM-plotter platform, which integrates transcriptomic and clinical data from large patient datasets. Notably, these datasets report total miR-210 expression without strand-specific resolution; thus, the observed associations are attributed to miR-210, with miR-210-3p being the predominant and best-characterized functional strand [8, 35]. This approach provides long-term patient follow-up (median 94 months) offering enhanced statistical power over shorter-term datasets such as TCGA (median: 25 months). High miR-210 expression or low *ISCU* levels were significantly associated with reduced overall survival in triple-negative (TNBC) and basal-like breast cancers, the most aggressive breast cancer subtypes (Fig. 8a and b). A weaker but still significant association was also observed in Luminal A and Luminal B tumors, while no prognostic value was detected in the HER2⁺/ER⁻ subgroup (Fig. 8c).

Importantly, this adverse prognostic pattern extended beyond breast cancer, with similar associations observed in hepatocellular carcinoma, pancreatic adenocarcinoma, and sarcoma (Fig. S2). Notably, these tumors displayed predominant enrichment of MCC-related genes rather than classical HIF target genes, reinforcing the relevance of the miR-210-3p-FOXM1-MCC axis in driving aggressive tumor phenotypes in distinct cancer types.

## Discussion

This study uncovers a previously unrecognized role of miR-210-3p in the regulation of mitotic gene expression and proliferative dynamics in solid tumors. While miR-210-3p has long been established as a canonical hypoxamiR, transcriptionally induced by HIFs under low oxygen conditions and best known for repressing genes involved in mitochondrial respiration and oxidative phosphorylation (e.g., *ISCU1/2, NDUFA4, SDHD*) [10, 38, 45, 46], its biological functions extend well beyond metabolic adaptation. Accumulating evidence has associated miR-210-3p with diverse oncogenic processes, including DNA repair inhibition, angiogenesis, immune evasion, and metastatic spread [13, 15, 47–51].

Our findings expand this landscape by revealing that miR-210-3p also promotes the activation of FOXM1 and associated mitotic gene expression program, independently of hypoxia or classical HIF target activation. Although previous studies have reported alterations in cell cycle regulation, centrosome amplification, and dysregulation of mitotic genes in association with miR-210-3p, these observations have remained largely descriptive and mechanistically disconnected [32–34]. Through pan-cancer transcriptomic analyses, we show that this mitotic module is not universally upregulated across tumors, but is instead enriched in select malignancies, including breast, liver, pancreatic, and soft tissue cancers. In these contexts, high miR-210-3p expression, often accompanied by *ISCU* repression, correlates with elevated expression of *FOXM1, CENPE, BUB1B,* and *MKI67*, key regulators of mitotic progression and cell division [52]. Conversely, tumors dominated by classical hypoxia signatures exhibit reduced expression of these mitotic genes, underscoring a transcriptional divergence between miR-210-3p activity and HIF-mediated hypoxic responses.

This context dependency was further supported by functional validation across cell models. While miR-210-3p overexpression induced *BUB1B* expression in distinct breast cancer cell lines, including MCF7 and MDA-MB-231, no such effect was observed in SCC38 head and neck carcinoma cells, despite comparable miR-210-3p levels. These findings reinforce the notion that miR-210-3p-mediated activation of the mitotic program is restricted to specific cellular contexts, in agreement with the tumor-specific transcriptional patterns observed in our pan-cancer analyses.

Functional studies in MCF7 breast cancer cells confirmed the transcriptional link between miR-210-3p and the mitotic program. Overexpression of miR-210-3p upregulated *FOXM1* and its downstream targets (*CENPE* and *BUB1B*) while stabilizing HIF1α and HIF2α under normoxic conditions, suggesting a feed-forward loop that sustains HIF signaling and promotes mitotic reprogramming. Notably, this effect differs from the hypoxic response, in which HIF1α activation is transient and subject to feedback regulation. In contrast, miR-210-3p overexpression leads to sustained HIF stabilization, which may contribute to the persistent activation of mitotic signaling observed in our system.

Mechanistically, silencing HIF1α (but not inhibiting HIF2α) abolished the miR-210-3p-induced *BUB1B* overexpression, indicating a specific requirement for HIF1α. These data support a model in which miR-210-3p indirectly amplifies mitotic signaling through HIF1α-dependent activation of FOXM1. miR-210 has been previously linked to HIF regulation through pathways such as GPD1L-dependent modulation of prolyl hydroxylase activity [53]. However, no consistent inverse correlation between miR-210-3p and *GPD1L* expression was observed in the TCGA tumors, arguing against *GPD1L* repression as the sole mechanism of HIF1α stabilization. Alternative explanations may involve indirect effects of miR-210-3p on cellular metabolism. For example, miR-210-3p is known to reprogram cellular metabolism (e.g. via *ISCU* inhibition), which could alter tricarboxylic acid (TCA) cycle intermediates (such as α-ketoglutarate/succinate levels) and thereby reduce prolyl hydroxylase (PHD) activity, leading to HIF stabilization [54]. In addition, feedback mechanisms within hypoxia signaling networks may further contribute to sustained HIF accumulation. At the downstream level, our data supports a functional link between miR-210-3p-HIF1α and FOXM1, which drive the mitotic transcriptional program. However, we cannot exclude that additional effectors contribute to the observed phenotypes. FOXM1 and its target genes, including *BUB1B* and *CENPE*, are well-established regulators of mitotic fidelity and proliferation, and their dysregulation is sufficient to impair cell division. Further studies will be required to delineate the precise molecular intermediates linking miR-210-3p expression to HIF stabilization and to dissect the individual contribution of FOXM1, CENPE and BUB1B and other effectors to the phenotypic outcomes described here.

Paradoxically, miR-210-3p overexpression impaired cell proliferation despite activating mitotic genes. Cells overexpressing miR-210-3p exhibited pronounced mitotic delays, cytokinesis failure, chromosome misalignment, and spindle defects, consistent with mitotic stress and supported by combined morphological and quantitative analyses. Cell cycle analysis revealed accumulation in G1/G0, reduced S-phase entry, and defective G2/M transition. Additionally, cells showed significantly enlarged nuclei, consistent with failed mitotic exit or arrest [55]. In contrast, hypoxia did not mimic these effects: hypoxic exposure transiently elevated pFOXM1 but failed to sustain mitotic gene induction, instead modestly increasing proliferation. By contrast, normoxic miR-210-3p overexpression produced sustained HIF activation and persistent induction of FOXM1 and its targets (*BUB1B*, *CENPE*). These findings highlight miR-210-3p’s unique ability to drive a mitotic transcriptional program that is mechanistically distinct from the broader hypoxic response. Thus, miR-210-3p does not simply mimic hypoxia but rather uncouples HIF activation from the general hypoxic response, selectively amplifying HIF1α-dependent mitotic signaling. This uncoupling may explain why miR-210-3p induces aberrant mitosis and impaired proliferation, whereas hypoxia elicits a coordinated adaptive response.

Overall, our findings reveal a dual, context-dependent role for miR-210-3p in cancer cells. While miR-210-3p induces the expression of mitotic regulators, its overexpression disrupts mitotic fidelity and impairs proliferation in vitro. This may reflect a threshold effect, whereby excessive FOXM1 activation destabilizes mitotic control, shifting cells from coordinated division toward mitotic stress and chromosomal instability. In this context, such alterations could facilitate the emergence of more aggressive cellular subclones and contribute to tumor progression in vivo. This framework may help reconcile previous observations in which miR-210-3p suppresses proliferation in vitro [21, 22, 56] yet is consistently associated with poor prognosis and aggressive tumor phenotypes in patients [57, 58].

Clinically, this transcriptional divergence is reflected in patient data. High miR-210-3p expression (or *ISCU* loss) correlates with poorer survival in triple-negative/basal-like breast cancer, as well as in hepatocellular, pancreatic, and sarcoma tumors. These cancers preferentially express FOXM1 and mitotic checkpoint genes rather than classic HIF targets, underscoring the clinical relevance of the miR-210-3p-FOXM1-mitotic axis in driving aggressive tumor biology.

This study has several limitations. First, although the pan-cancer analyses support a relevance of the miR-210-3p-associated mitotic program in some tumor types, most mechanistic experiments were performed in MCF7 breast cancer cells, and therefore the extent to which these findings can be generalized to other tumor types remains to be established. Second, although FOXM1 and its downstream targets *BUB1B* and *CENPE* were consistently associated with the observed phenotypes, the individual contribution of each component to mitotic defects and proliferation arrest was not directly dissected. Finally, the study relies primarily on in vitro models and transcriptomic associations, and therefore the impact of the miR-210-3p-HIF1α-FOXM1 axis on tumor evolution and clonal selection in vivo remains to be determined. Future studies addressing these questions will further refine the mechanistic framework proposed here.

In summary, we identify miR-210-3p as a key modulator of mitotic gene expression and cell cycle regulation in cancer. By promoting FOXM1 activation, HIF stabilization, and mitotic reprogramming, miR-210-3p reshapes the proliferative and transcriptional landscape of tumor cells. This dual role, as both a transcriptional driver and a disruptor of mitotic fidelity, positions miR-210-3p as a context-dependent regulator of tumor progression, with potential as a biomarker and therapeutic target in malignancies characterized by pseudohypoxia and mitotic dysregulation.

## Material and methods

### Cell culture and treatments

MCF7 (breast cancer), SCC38 (squamous cell carcinoma), MDA-MB-231 (breast cancer) and SW13 (adrenocortical carcinoma) cell lines were maintained in Dubelcco’s modified Eagle’s medium (DMEM; Gibco, Thermo Fisher Scientific, Waltham, MA, USA) supplemented with 10% fetal bovine serum (FBS; Gibco, Thermo Fisher Scientific), 100 units/mL penicillin (Gibco, Thermo Fisher Scientific), 100 µg/mL streptomycin (Gibco, Thermo Fisher Scientific), 1% MEM Non-Essential Amino Acids Solution (Gibco, Thermo Fisher Scientific), 2 mM L-glutamine (Gibco, Thermo Fisher Scientific) and plasmocin (InvivoGen, San Diego, CA, USA) at a prophylactic concentration of 2.5 µg/mL. Pheochromocytoma (PCC)-derived primary cells were cultured in DMEM/F12 (Gibco, Thermo Fisher Scientific) supplemented with 15% FBS, 100 units/mL penicillin, 100 µg/mL streptomycin, 2 mM L-glutamine, 1% B-27 and N-2 (Thermo Fisher Scientific), 10 SB-202190 (Sigma-Aldrich, St. Louis, MO, USA), 1 mM N-acetylcysteine (Sigma-Aldrich), 10 µM Y-27632 (Tocris, Bristol, UK), 500 nM A-8301 (Tocris), 50 ng/mL each of epidermal growth factor (EGF) and fibroblast growth factor (FGF) and 100 ng/mL IGF (StemCell Technology, Vancouver, BC, Canada). Cells were cultured under normoxic conditions (21% O₂, 5% CO₂, 37 °C) or exposed to hypoxia (1% O_2_, 5% CO₂) using a Heracell 150 incubator. Where specified, cells were treated with 1 µM nocodazole (Sigma-Aldrich) for 14 h or 2 µM thiostrepton (Tocris) for 24 h. To chemically induce HIF stabilization under normoxia, cells were treated for 24 h with 1 mM dimethyloxalylglycine (DMOG; MedChemExpress, Monmouth Junction, NJ, USA) or 150 µM deferoxamine mesylate (Sigma-Aldrich). PT2385 (MedChemExpress, New Jersey, USA) was used to block HIF2α activity. Stock solution was prepared at 10 mM in sterile DMSO. MCF7 cells were treated with 50 µM PT2385 for 18 h, in parallel with transfection of either miR-210-3p mimics or a non-targeting control. Control cells were treated with dimethyl sulfoxide (DMSO; Sigma-Aldrich), used as solvent at equal volumes to those of compound-treated conditions. All cell lines were routinely tested for mycoplasma contamination using the MycoAlert™ Mycoplasma Detection Kit (Lonza, Basel, Switzerland).

### Cell transfection and generation of stable knockdown

Cells (1 × 10^4^) were seeded in 6-well plates and transfected with either a mirVana™ miRNA non-targeting miRNA control or hsa-miR-210-3p mirVana™ miRNA mimic (Thermo Fisher Scientific) using Lipofectamine™ RNAiMAX Transfection Reagent (Thermo Fisher Scientific) according to the manufacturer’s instructions. Unless otherwise indicated, miR-210-3p mimics were used at a final concentration of 5 nM. For *HIF1A* gene silencing, a non-targeting short hairpin RNA (shRNA) control and six independent *HIF1A*-targeting shRNA constructs cloned into the pGIPZ lentiviral vector were obtained from Horizon Discovery Ltd. (Cambridge, UK). Lentiviral particles were generated by transfecting HEK293 T cells with the shRNA plasmids together with packaging plasmids (VSV-G and psPAX2). Viral supernatants were collected 48 h post-transfection. MCF7 cells were incubated with viral supernatants in the presence of Polybrene (8 µg/mL). After 48 h, cells were washed and selected in medium containing puromycin (4 µg/mL) for 10 days.

### Cell cycle assay

Cells were fixed in cold 70% (vol/vol) ethanol 16 h at −20ºC, treated with RNase A (1 mg/mL, Thermo Fisher Scientific) for 5 min at room temperature, and stained with propidium iodide (PI, 1 mg/mL, Sigma-Aldrich) for 30 min at 4ºC. Samples were analyzed by flow cytometry using a FACS Aria IIu (BD biosciences, San Jose, CA, USA), and 20,000 events were collected per sample. Data were analyzed using FlowJo software (BD Biosciences).

### Proliferation assay

Cells were seeded in 12-well plates (1.2 × 10^4^ cells/well, in triplicates per condition) and harvested at specified time points. Cell proliferation was quantified by manual counting using a Neubauer chamber.

### Colony formation assay

Cells were seeded at low density in 12-well plates (200 cells per well for SCC38 and MCF7 cell lines, and 400 cells per well for MDA-MB-231) and cultured for 7 days. Colonies were fixed with ice-cold methanol and stained with 0.1% crystal violet. Colony number and area were quantified using an ImageJ plugin.

### Apoptosis analysis

Apoptosis was assessed by Annexin V/7-AAD staining and mitochondrial membrane potential analysis. For Annexin V assays, cells were harvested, washed, and resuspended in Annexin V binding buffer (BioLegend, San Diego, CA, USA) at 2.5 × 10⁶ cells/mL, followed by incubation with fluorochrome-conjugated Annexin V and 7-AAD according to the manufacturer’s instructions. Samples were analyzed by flow cytometry (CytoFLEX S, Beckman Coulter), acquiring 20,000 events per sample. Mitochondrial membrane potential was evaluated using DiOC6(3) staining in combination with PI. Briefly, cells were incubated with DiOC6(3) (final concentration 20 nM, Sigma-Aldrich) for 25 min at 37 °C in complete DMEM, followed by PI staining immediately prior to acquisition. Samples were analyzed by flow cytometry under identical acquisition settings and processed using the FlowJo software (BD Biosciences) and CytoFLEX (Beckman Coulter).

### RNA extraction and RT–qPCR

Total RNA was extracted using mirVana RNA isolation kit (Invitrogen, Thermo Fisher Scientific) following manufacture’s instructions. First-strand cDNA was synthesized with the Maxima First Strand cDNA synthesis kit (Thermo Fisher Scientific). Quantitative PCR was performed using TaqMan™ PCR Master Mix and gene-specific TaqMan™ probes (Applied Biosystems, Foster City, CA, USA) on a StepOne™ Real-Time PCR system (Applied Biosystems). Cyclophilin A was used as the endogenous control for mRNA quantification, and U6 snRNA for miR-210-3p expression. Relative expressions were calculated using the 2^−∆∆CT^ method. *ISCU* and *HIF1A* mRNA levels were measured using SYBR™ Green PCR Master Mix (Applied Biosystems) with the following primers: *ISCU*: 5’-CCTCCAGCTCATTAGCCACT-3’ and 5’-CTTCCTCCACCGTCTTCTTTCCT-3’; *HIF1A*: 5'-CTGAAGACACAGAAGCAAAGAACC-3’ and 5'-CAAGTCTAAATCTGTGTCCTGAGTAGAAA-3’; *PPIA*: forward: 5’-CATCTGCACTGCCAAGACTGA-3’ and reverse: 5’-TTGCCAAACACCACACCACATGCTT-3’.

### Immunofluorescence and time-lapse microscopy

Cells were seeded at 70%–80% confluency in µ-Plate 96-well square-bottom plates (Ibidi GmbH, Gräfelfing, Germany) and fixed with 4% paraformaldehyde for 15 min at room temperature. After washing, cells were permeabilized and blocked in PBS supplemented with 1% BSA and 0.3% Triton X-100. Primary antibodies were incubated overnight at 4 °C in blocking buffer. The following primary antibodies were used: anti-CENPE (Abcam, Cambridge, UK) at 1:200 dilution and anti-α-tubulin (ProteinTech, Chicago, IL, USA) at 1:500 dilution. Cells were then incubated for 2 h at room temperature with the appropriate secondary antibodies: Alexa Fluor™ Plus 555-conjugated goat anti-rabbit IgG (Thermo Fisher Scientific) or Alexa Fluor™ 488-conjugated goat anti-mouse IgG (Thermo Fisher Scientific), both diluted 1:500. Nuclei were counterstained with DAPI (Ibidi GmbH). Fluorescence images were acquired using a Zeiss AxioObserver Z1 microscope. For live-cell imaging, cells were cultured in 6-well µ-slides (Ibidi GmbH) under standard conditions and imaged using the same Zeiss AxioObserver Z1 system equipped with a stage-top incubator to maintain temperature, humidity, and CO₂. Time-lapse images were acquired every 5 min for up to 17 h using phase-contrast optics. The time elapsed from individual cell rounding (detachment) to complete cytokinesis of the two daughter cells was measured for individual mitotic events.

### Western blot

Cells at 80%–90% confluence were lysed in RIPA buffer (Sigma-Aldrich) supplemented with protease and phosphatase inhibitor cocktails (Sigma-Aldrich). Equal amount of proteins (50 μg) were fractionated by SDS-PAGE and transferred (120 V, 2 h) onto PVDF membranes (Bio-Rad Laboratories, Hercules, California, USA). Membranes were incubated with primary antibodies against pFOXM1 (Cell Signaling, Danvers, Massachusetts, USA; 1:300), HIF1α (Novus Biologicals, Centennial, Colorado, USA; 1:200), HIF2a (Abcam; 1:200), CENPE (Abcam; 1:300) or a-tubulin (ProteinTech; 1:1,000). Bound antibodies were detected using IRDye 680 IgG (LI-COR Biosciences, Lincoln, NE, USA) secondary antibody (1:10,000) and visualized using the Odyssey Fc Imaging System (LI-COR Biosciences). Densitometric quantifications were performed using Image Studio software (LI-COR Biosciences).

### Statistical and bioinformatic analysis

To assess miR-210-3p expression across human cancers, we used the TNMplot platform [59] which integrates uniformly processed transcriptomic data from GEO, GTEx, and TARGET repositories. The dataset comprises 56,090 tissue samples, including 5,648 normal and 40,442 tumor samples. Expression values were normalized using robust multi-array average (RMA) method. For independent validation and correlation analysis, we retrieved miRNA and RNA-seq data from TCGA database (https://portal.gdc.cancer.gov/projects), comprising 8,566 primary tumor samples across 21 solid tumor types. To define the transcriptomic programs associated with miR-210-3p activation, we performed Pearson correlation analysis between miR-210-3p and all protein-coding genes across tumor types. Genes were considered significantly associated if they showed strong positive (R > 0.40) or negative (R < –0.40) correlations (adjusted p < 0.001) in at least five tumor types. Differential associations patterns were further analyzed to characterize context-specific transcriptional outputs of miR-210-3p across cancers. Functional enrichment analysis was performed using Gene Ontology (GO Biological Process) and pathway annotations from KEGG, Reactome, and PID databases. To reduce redundancy among enriched terms, functionally related GO categories were grouped based on shared biological meaning, and a representative term was selected to summarize each functional category identified in the pan-cancer analysis. Subsequently, gene set enrichment analysis was carried out independently for each tumor type. From these analyses, we focused on terms that were significantly enriched and that overlapped with the predefined functional categories identified at the pan-cancer level. Putative miR-210-3p targets were identified using TargetScan, and MSigDB miRNA-target prediction modules. For survival analysis, we used the KMplot database [60]. The METABRIC dataset (n > 1,900), which includes long-term follow-up (median: 94 months) was used for analysis of breast cancer patients [61]. Overall survival was compared between patients with high and low miR-210-3p or *ISCU* expression groups using the log-rank test and the KM plotter platform [60, 62]. The optimal expression cutoff was automatically determined by the tool based on the best performing threshold. All graphs and statistical analysis were performed using either the statistical software R (v.4.0.2) with the ggplot2 package, or GraphPad Prism 8.0 software (GraphPad Software, La Jolla, CA, USA). Statistical significance for experimental data was assessed using unpaired two-tailed Student’s t-tests, with p < 0.05 considered statistically significant.

## Supplementary Information

Supplementary Material 1.

Supplementary Material 2.

Supplementary Material 3.

Supplementary Material 4.

Supplementary Material 5.

Supplementary Material 6.

Supplementary Material 7.

## Data Availability

The datasets during and/or analyzed during the current study available from the corresponding author on reasonable request.
