# Supplementary material for: A dual role for miR-210-3p in hypoxia-driven signaling and mitotic regulation in cancer
Source: Mol Biomed. 2026 Aug 10;7:134. doi: 10.1186/s43556-026-00530-4 (PMC13454091; doi:10.1186/s43556-026-00530-4)
Supplement: Supplementary file 1 [file 43556_2026_530_MOESM1_ESM.docx]

**Supplementary Material**

**A dual role for miR-210-3p in hypoxia-driven signaling and mitotic regulation in cancer**

Jaime San-Juan-Guardado^1,2^, María Turienzo-Durán^1^, Álvaro Suárez-Priede^1,2^, Nerea Gómez-Suárez^1,2^, Candelaria Aguilar-García^1,2,3^, Tamara Cubiella^1,2,*^ and María-Dolores Chiara^1,2,*^

^1^ Health Research Institute of the Principality of Asturias, 33011 Oviedo, Spain.

^2^ Institute of Oncology of the Principality of Asturias, University of Oviedo, 33006 Oviedo, Spain.

^3^ Department of Functional Biology, Immunology, University of Oviedo, 33006 Oviedo, Spain

**Correspondence to**:

María-Dolores Chiara

Health Research Institute of the Principado de Asturias

Av del Hospital Universitario s/n

33011-Oviedo

Spain

mdchiara.uo@uniovi.es

Tel.: +34 985107956

Tamara Cubiella

Health Research Institute of the Principado de Asturias

Av del Hospital Universitario s/n

33011-Oviedo

Spain

tamara.cubiella@ispasturias.es

Tel.: +34 985107956

* Corresponding authors

**Contents**

**Fig. S1. Densitometric quantification of the Western blot analyses shown in Figures 4–6.**

**Fig. S2. Prognostic impact of miR-210 and *ISCU* expression in multiple tumor types.**

**Video 1.** Time-lapse MCF7 Ct cells

**Video 2.** Time-lapse MCF7 miR cells

**Supplementary Tables**

**Table S1.** miR-210-3p-mRNA expression correlations in primary tumor samples

**Table S2**. Pan-cancer functional enrichment of genes negatively and positively correlated with miR-210-3p

**Table S3**. Tumor-specific functional enrichment of genes negatively correlated with miR-210-3p expression

**Table S4**. Tumor-specific functional enrichment of genes positively correlated with miR-210-3p expression

**Fig. S1.** Protein band intensities were quantified using Image Studio Software (LI-COR Biosciences) and normalized to the corresponding loading control (α-tubulin). Data is presented as fold change relative to the indicated control condition in each experiment. Each dot represents an independent biological replicate, and bars indicate mean ± SEM. (**a, b**) Quantification of the Western blots shown in Fig. 4g left (a) and right (b). (**c, d**) Quantification of the Western blots shown in Fig. 4h (c) and Fig. 4i (d). (**e, f**) Quantification of the Western blots shown in Fig. 5c (e) and Fig. 5d (f). (**g**) Quantification of the Western blots shown in Fig. 6d.

**Fig. S2.** Kaplan-Meier survival analyses showing the association between high miR-210 expression (top row) or low *ISCU* expression (bottom row) and overall survival in sarcoma (SARC), pancreatic adenocarcinoma (PAAD), and liver hepatocellular carcinoma (LIHC) using TCGA data. Patients were stratified into “Yes” (red lines: high miR-210 or low *ISCU* expression) and “No” (blue lines: low miR-210 or high *ISCU* expression) groups. p-values are from the log-rank test.
